# Supplementary material for: Efficient RNA drug delivery using red blood cell extracellular vesicles
Source: Nat Commun. 2018 Jun 15;9:2359. doi: 10.1038/s41467-018-04791-8 (PMC6004015; doi:10.1038/s41467-018-04791-8)
Supplement: Supplementary file 1 — Supplementary Information [file 41467_2018_4791_MOESM1_ESM.pdf]

Supplementary Figures for “Efficient RNA drug delivery using red blood cell extracellular vesicles” by Usman et al.

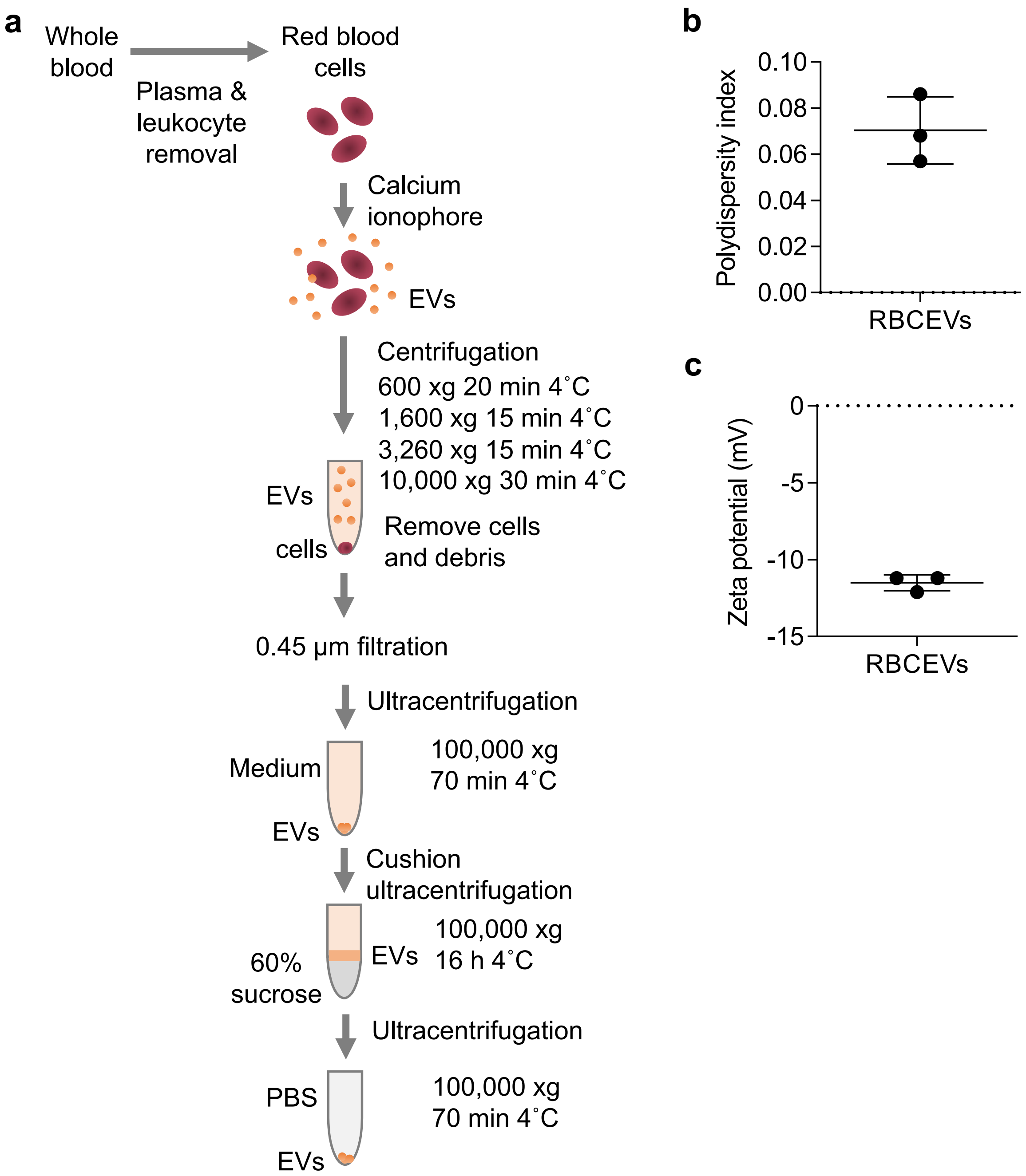

Supplementary Figure 1| Purification and characterization of extracellular vesicles (EVs) from human red blood cells (RBCs). (a) Purification method: culture supernatants were collected from ionorphore-treated human red blood cells and subjected to multiple steps of low speed centrifugation to remove cells and debris. EVs were purified by 3 rounds of ultracentrifugation including one with 60% sucrose cushion at 100,000 xg. **(b)** Polydispersity index and **(c)** Zeta potential of RBCEVs from 3 donors were determined by using a Zetasizer Nano (mean ± SEM).

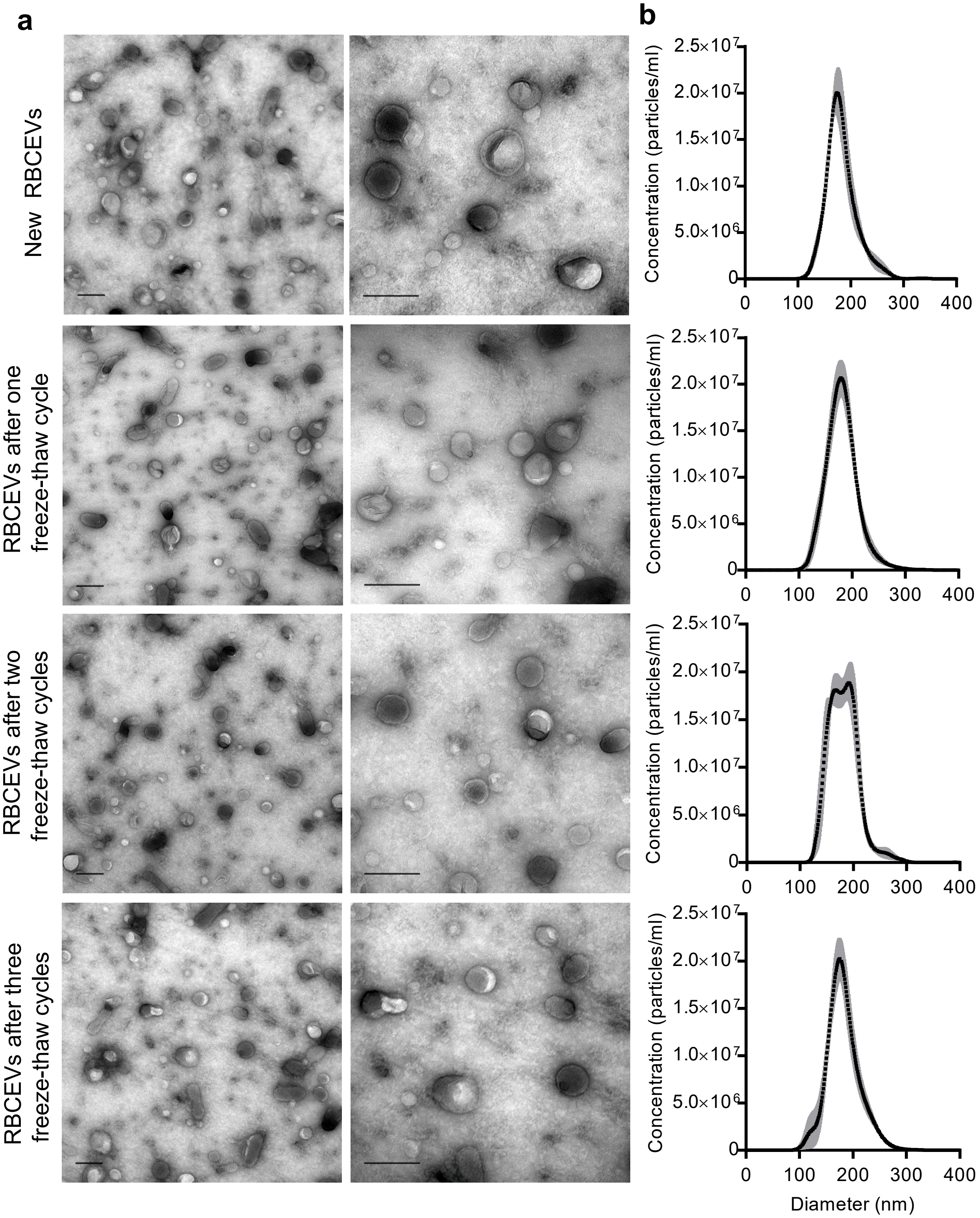

Supplementary Figure 2| Morphology and size distribution of RBCEVs after multiple freeze-thaw cycles. (a) Representative transmission electron microscopy images of RBCEVs from the same batch after 1-3 freeze-thaw cycles. Images were captured at 42000X (left) and 86000X (right). Scale bar, 200 nm. **(b)** Average concentrations of RBCEVs (100,000x dilution) from three donors ± SEM (grey) and their size distribution, determined using a Nanosight analyzer, after 1-3 freeze-thaw cycles.

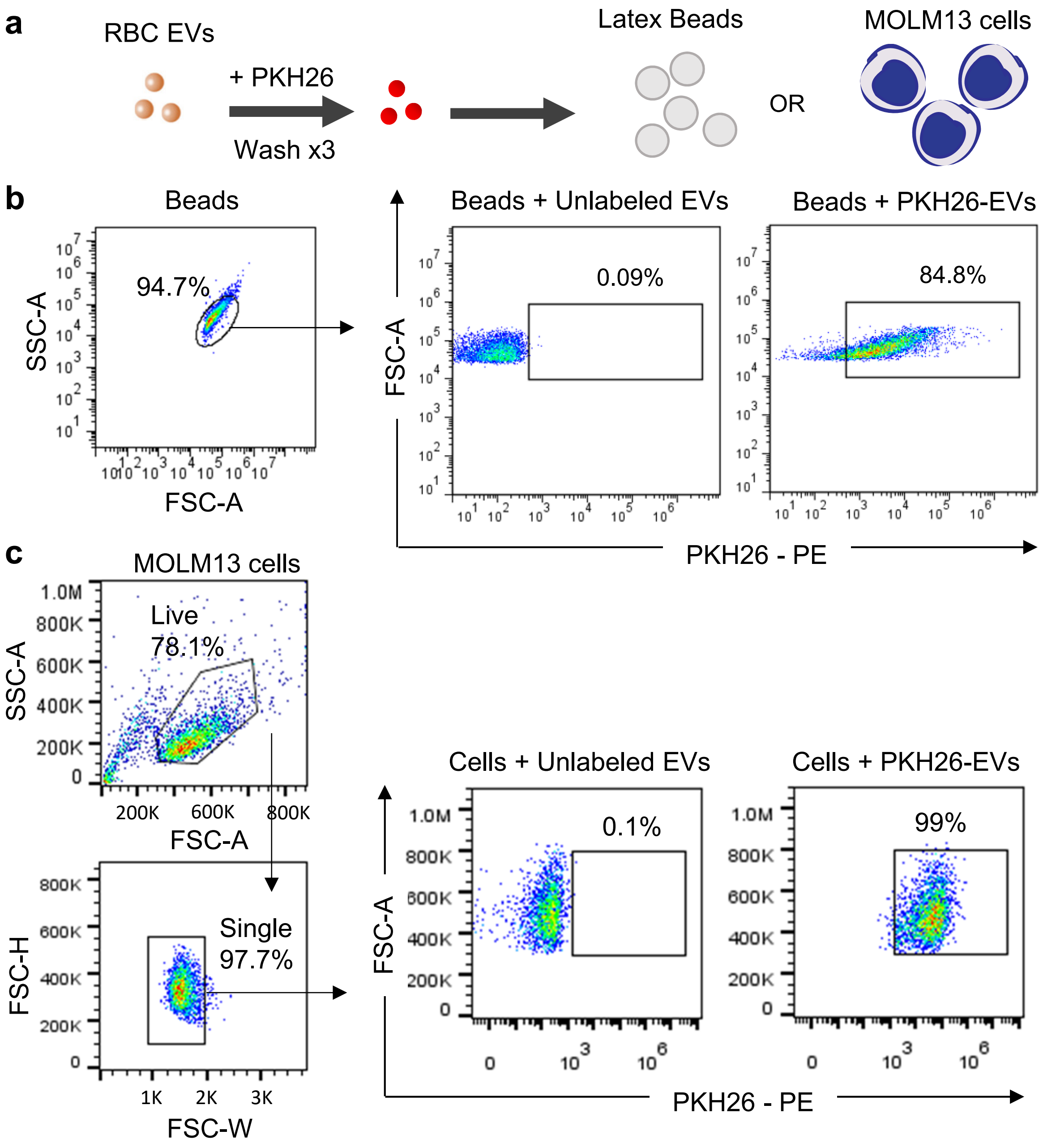

Supplementary Figure 3| RBCEVs are taken up by leukemia MOLM13 cells. (a) Schematic presentation of the EV uptake assay: RBCEVs were labeled with PKH26 (a red fluorescent membrane dye), washed three times using ultracentrifugation and incubated with latex beads overnight or with MOLM13 cells for 24 hours. **(b)** FACS analysis of latex beads incubated with 12.4 x 10^{11} unlabeled or PKH26-labeled RBCEVs. The beads were gated based on forward scattering area (FSC-A) and size scattering area (SSC-A). The PKH26 fluorescence (PE channel) was plotted vs. FSC-A. Percentages of PKH26 positive beads are indicated above the gates. **(c)** FACS analysis of MOLM13 cells incubated with 12.4 x 10^{11} unlabeled or PKH26-labeled RBCEVs. The cells were gated based on FSC-A vs. SSC-A (live population) and FSC-width vs. FSC-height (single cells). Percentages of PKH26 positive cells are indicated above the gates.

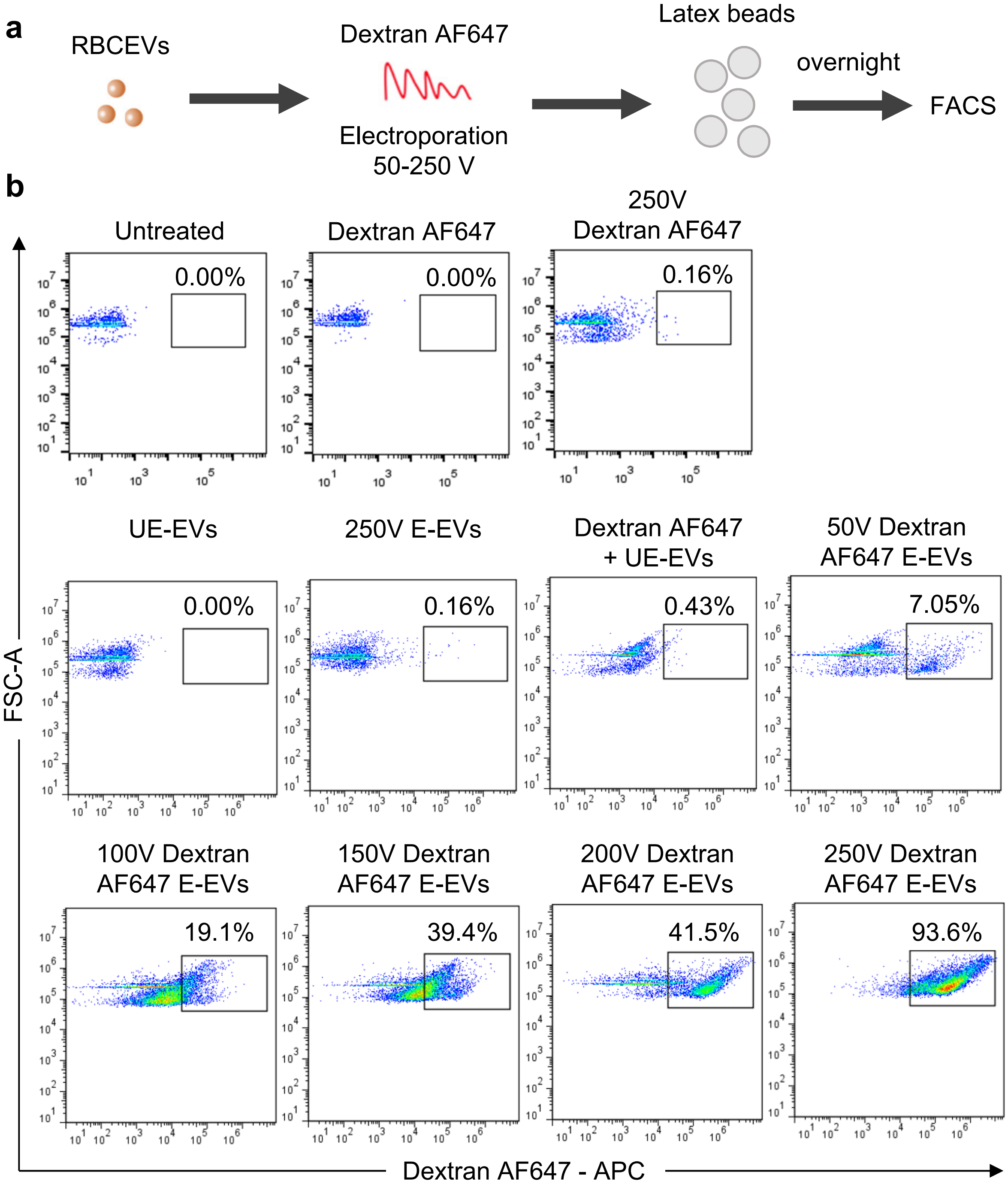

Supplementary Figure 4| Electroporation of RBCEVs with Dextran at different voltages. (a) Schematic presentation of EV electroporation: 8.25 x 10^{11} RBCEVs were mixed with 4 µg Alexa Fluor® 647 (AF647)-labeled Dextran and electroporated in OptiMEM at different voltages from 50 to 250 V. EVs were incubated with latex beads overnight and analyzed using FACS. **(b)** FACS analysis of AF647 fluorescence (APC channel) and FSC-A of the beads that were incubated with Dextran AF647, electroporated Dextran AF647, electroporated EVs (E-EVs) or unelectroporated EVs (UE-EVs). The percentages of AF647 positive beads are indicated above the gates.

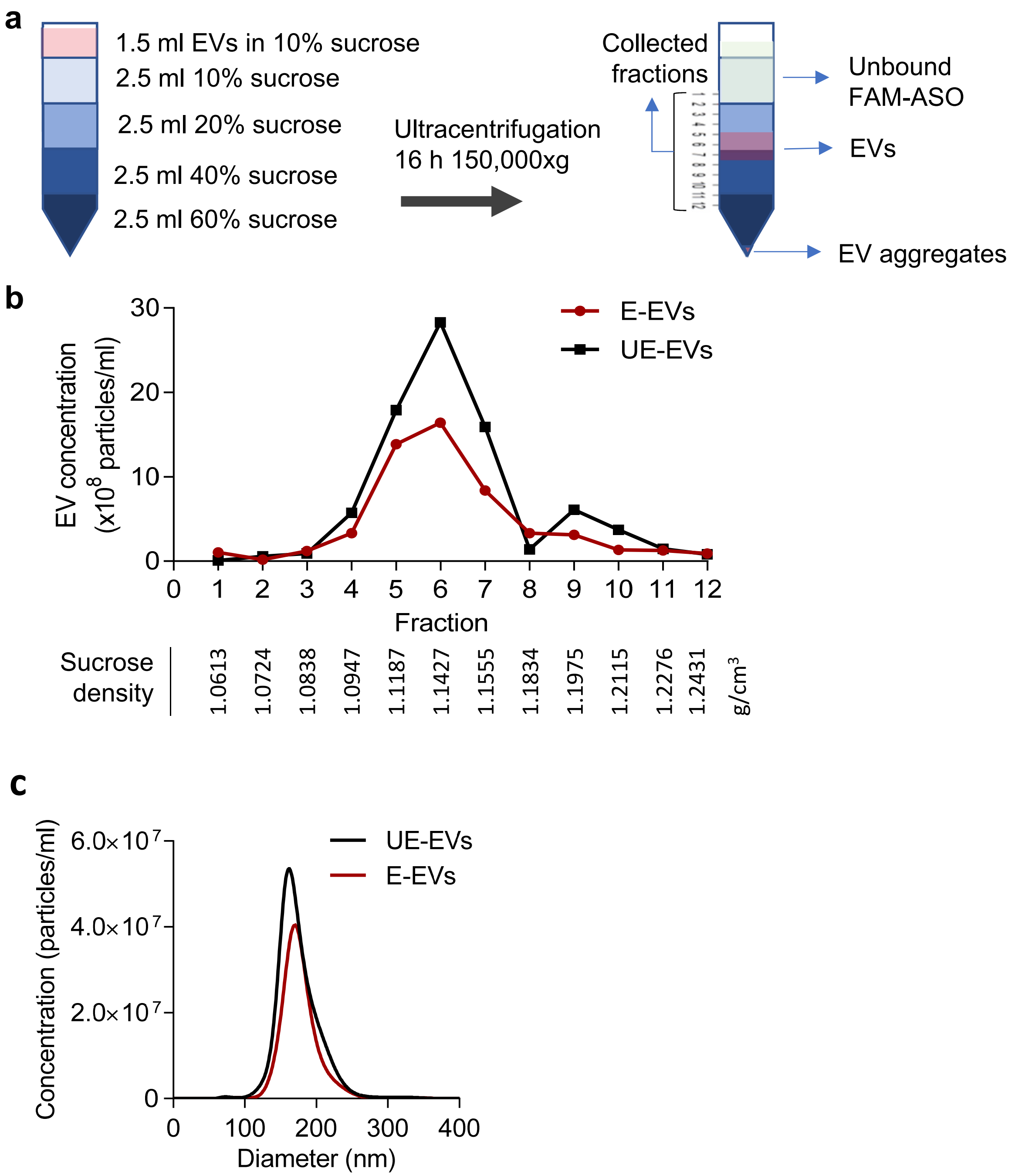

Supplementary Figure 5| Characterization of electroporated RBCEVs.
(a) Schema of top-down sucrose density gradient separation of RBCEVs. **(b)** Concentrations of unelectroporated (UE-EVs) or FAM-ASO-electroporated RBCEVs (E-EVs) in each sucrose fraction (200x dilution) were determined using a Nanosight analyzer and the density of sucrose was determined using a refractometer. **(c)** Size distribution of EVs in fraction 6, determined using the Nanosight particle analyzer.

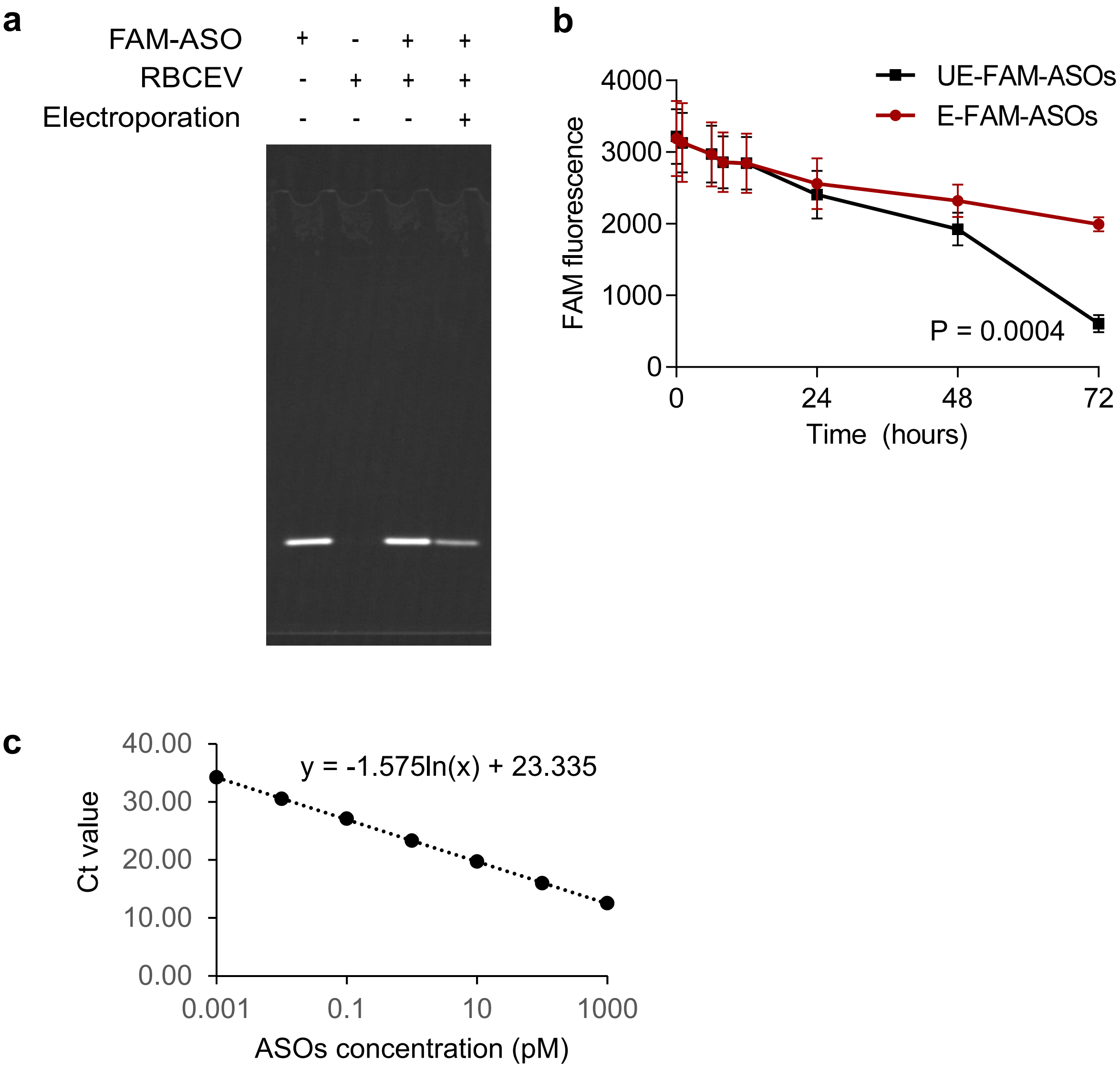

Supplementary Figure 6| Characterization of electroporated RBCEVs.
(a) FAM fluorescence of the unencapsulated FAM-ASOs from electroporated RBCEVs in 10% native gel. **(b)** Average fluorescent intensity of FAM-ASOs that were incubated with RBCEVs with or without electroporation (E or UE) in OptiMEM containing 50% FBS at 37ºC for 1-72 hours, determined using a Synergy™ microplatereader (mean ± SEM). P value was determined using Student's one-tail t-test (n = 3 independent repeats). **(c)** Standard curve of 125b-ASOs concentration vs. Ct values were determined using Taqman qRT-PCR.

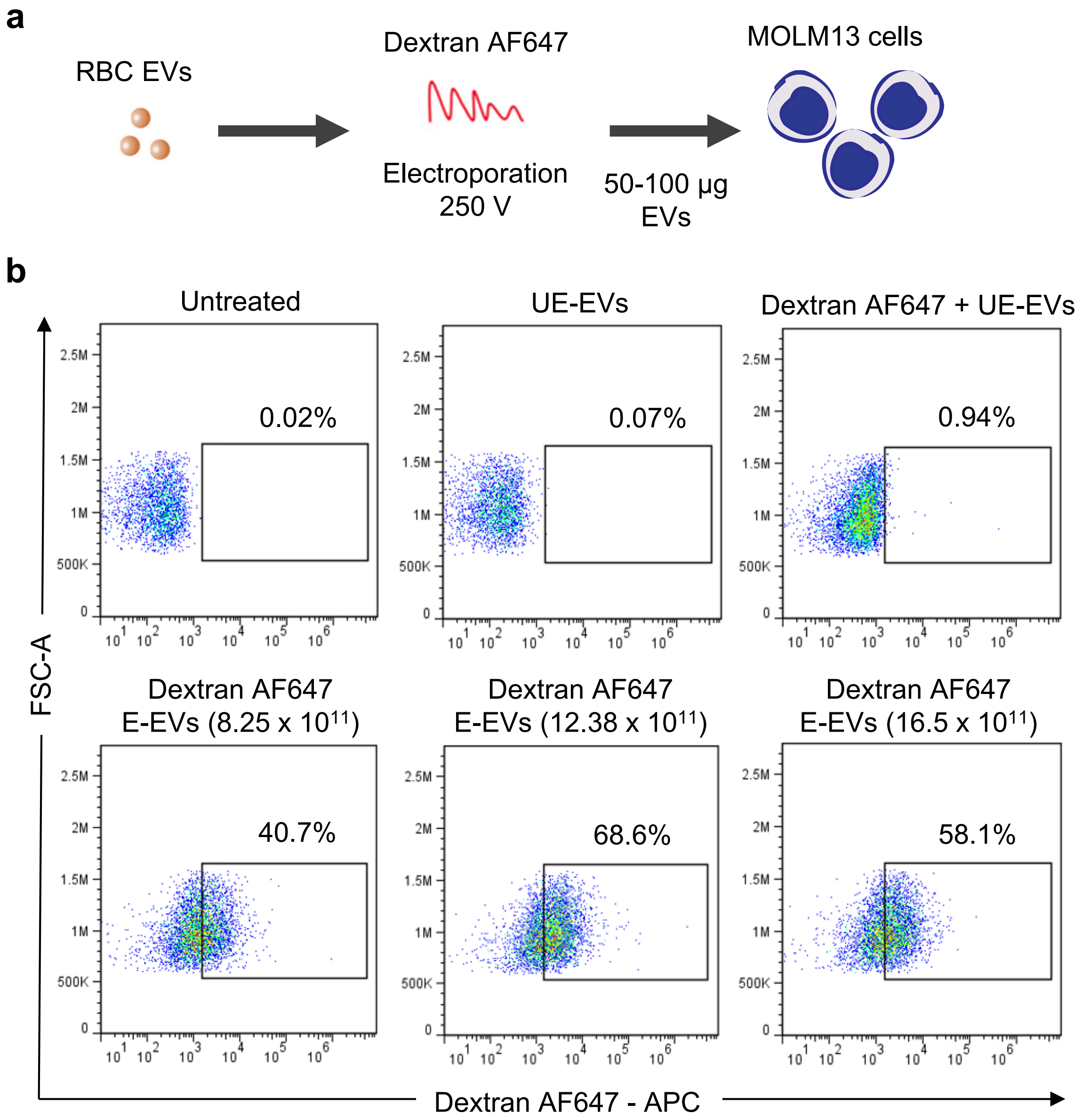

Supplementary Figure 7| RBCEVs deliver Dextran to leukemia MOLM13 cells.
(a) Schematic presentation of Dextran delivery: 8.25 – 16.5 x 10^{11} RBCEVs were mixed with 4 µg Dextran AF647 and electroporated at 250 V. Electroporated EVs were incubated with MOLM13 cells for 24 hours. **(b)** FACS analysis of Dextran AF647 fluorescence in MOLM13 cells that were untreated or incubated with 8.25 – 16.5 x 10^{11} Dextran-AF647 electroporated EVs (E-EVs) or 16.5 x 10^{11} unelectroporated (UE-EVs).

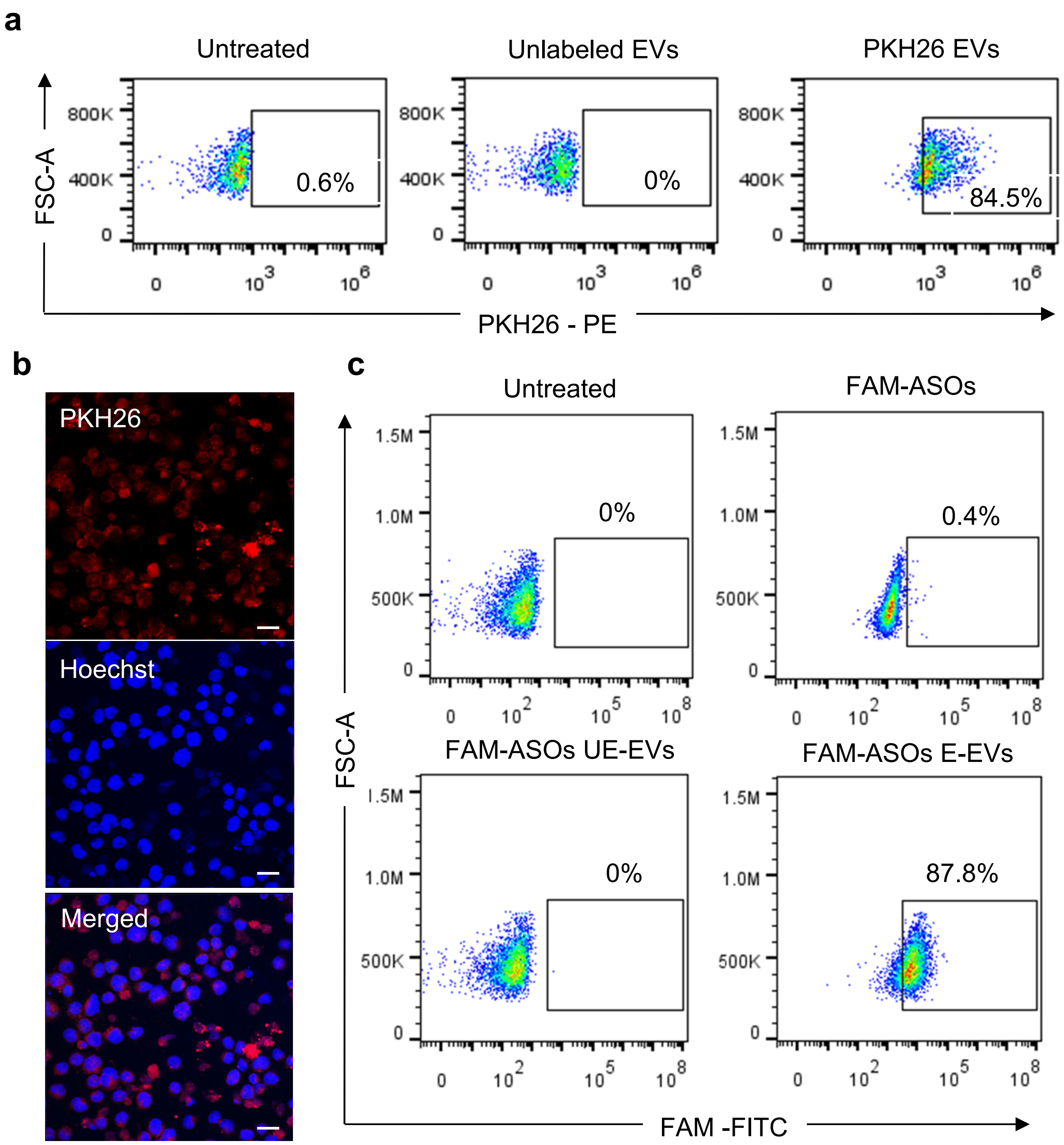

Supplementary Figure 8| RBCEVs deliver antisense oligonucleotides (ASOs) to leukemia NOMO1 cells. (a) FACS analysis of PKH26 (PE channel) in NOMO1 cells that were untreated or incubated with 12.4 x 10^{11} PKH26-labeled EVs. **(b)** Representative confocal microscopy images of NOMO1 cells treated with PKH26-labeled EVs. Scale bar, 20 μm. **(c)** FACS analysis of FAM fluorescence (FITC channel) in NOMO1 cells that were untreated or incubated with FAM-ASOs or with 12.4 x 10^{11} unelectroporated EVs (UE-EVs) or with FAM-ASOs-electroporated EVs (E-EVs).

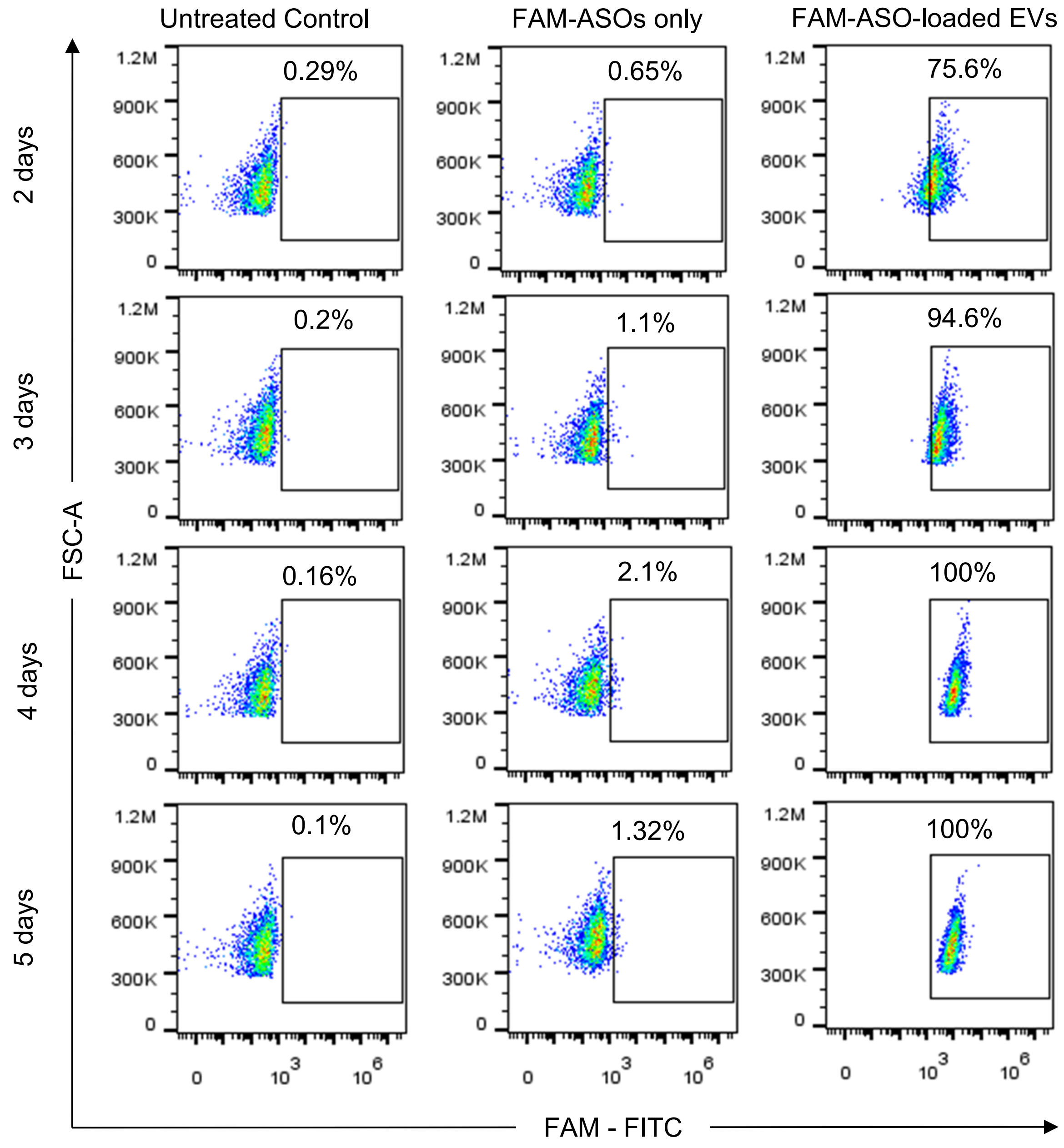

Supplementary Figure 9| Uptake of ASOs by leukemia cells over time. FACS analysis of FAM fluorescence (FITC channel) in MOLM13 cells that were untreated or incubated with 400 pmol FAM-ASOs alone or with 12 x 10^{11} FAM-ASO-electroporated RBCEVs for 5 days. The percentages of FAM positive cells are shown above the gates.

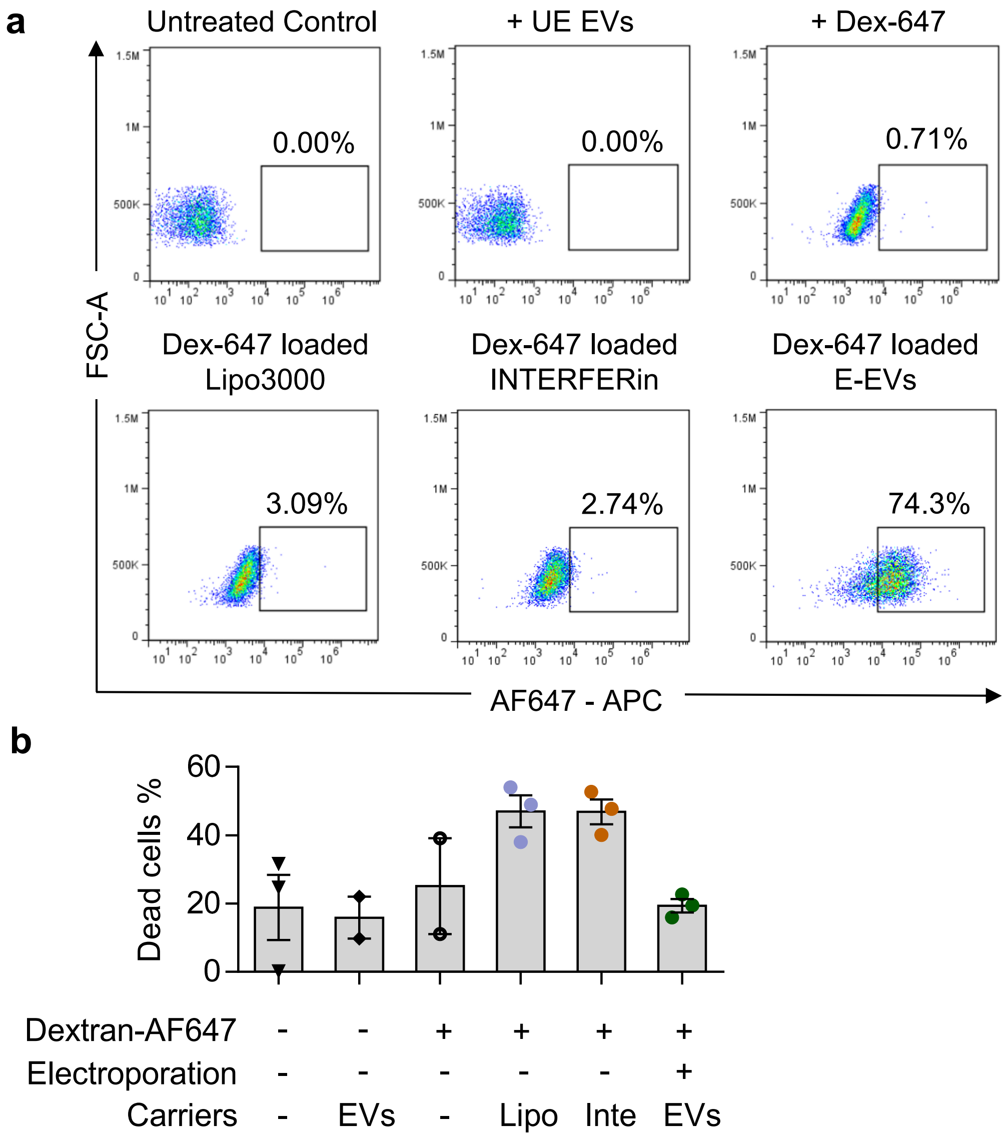

Supplementary Figure 10| RBCEVs confer higher efficiency and lower toxicity than Lipofectamine™ 3000 and INTERERin® in the delivery of Dextran to MOLM13 cells. (a) FACS analysis of AF647 fluorescence in MOLM13 cells that were untreated, incubated with unelectroporated RBCEVs (UE-EVs), with Dextran-AF647 (Dex-647) alone, with Dex-647 loaded Lipofectamin™ 3000 (Lipo3000), with Dex-647 loaded INTERFERin® or with 12.4 x 10^{11} Dex-647 electroporated RBC EVs (E-EVs) for 24 hours. **(b)** Percentage of dead cells determined using Propidium iodide (PI) staining in MOLM13 cells treated as in (a). The bar graph represents the average ± SEM of 2 to 3 repeats.

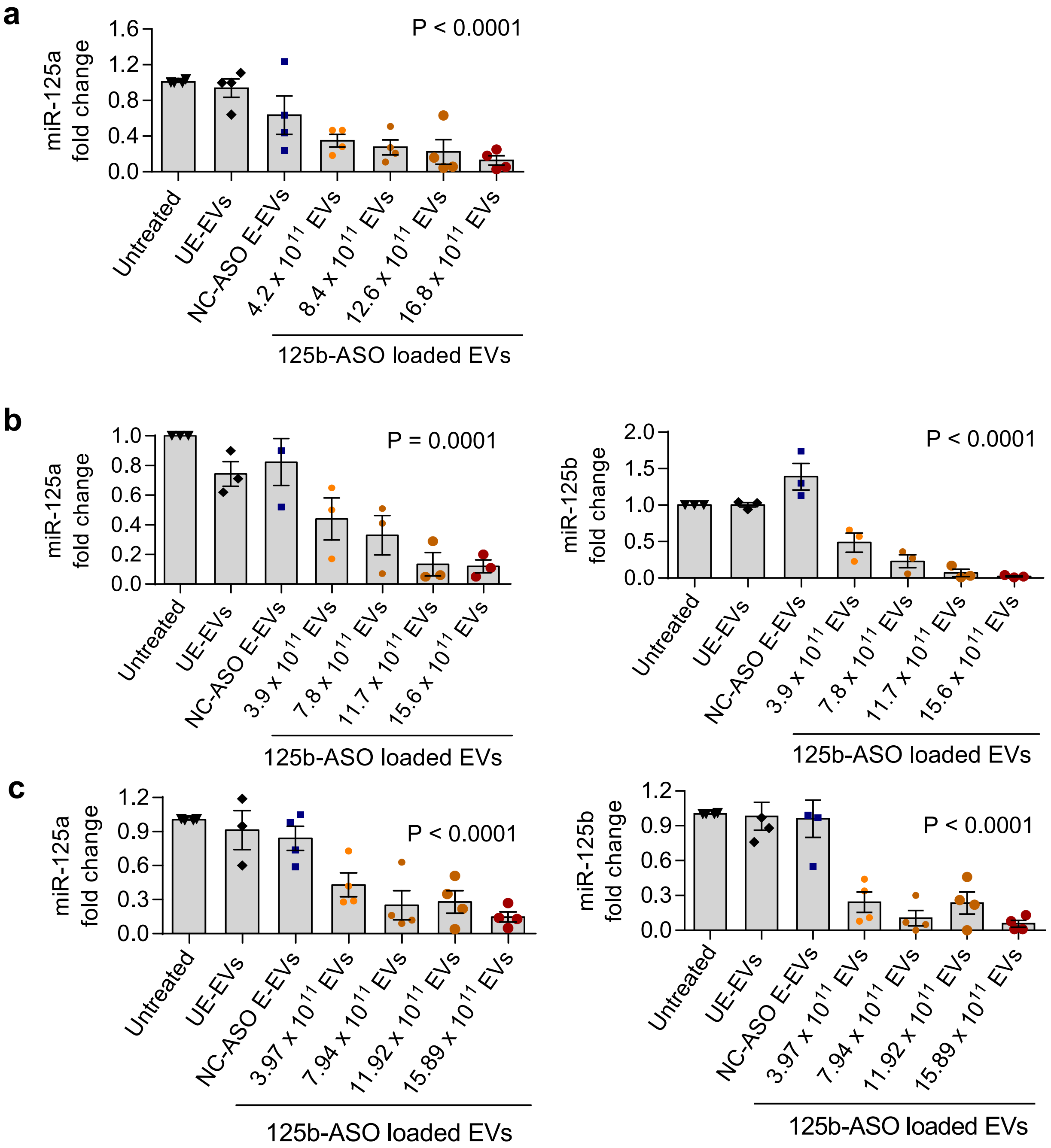

Supplementary Figure 11| Knockdown of the miR-125 family by EV-delivered ASOs in leukemia and breast cancer cells. (a) Expression fold change of miR-125a relative to U6b RNA in MOLM13 cells that were untreated, incubated with 16.8 x 10^{11} unelectroporated RBCEVs (UE-EVs), with 16.8 x 10^{11} NC-ASO electroporated RBCEVs (E-EVs) or 125b-ASO-electroporated RBCEVs at indicated doses for 72 hours. **(b)** Expression fold change of miR-125a and 125b, relative to U6b RNA, in NOMO1 cells treated with indicated doses of 125b-ASO-electroporated RBCEVs. **(c)** Expression fold change of miR-125a and 125b, relative to U6b RNA, in CA1a cells treated with indicated doses of 125b-ASO-electroporated RBCEVs. In all panels, miR-125a, 125b and U6b expression were determined using Taqman qRT-PCR in 3 or 4 cell passages (mean ± SEM). One-way Anova test result is shown in each graph.

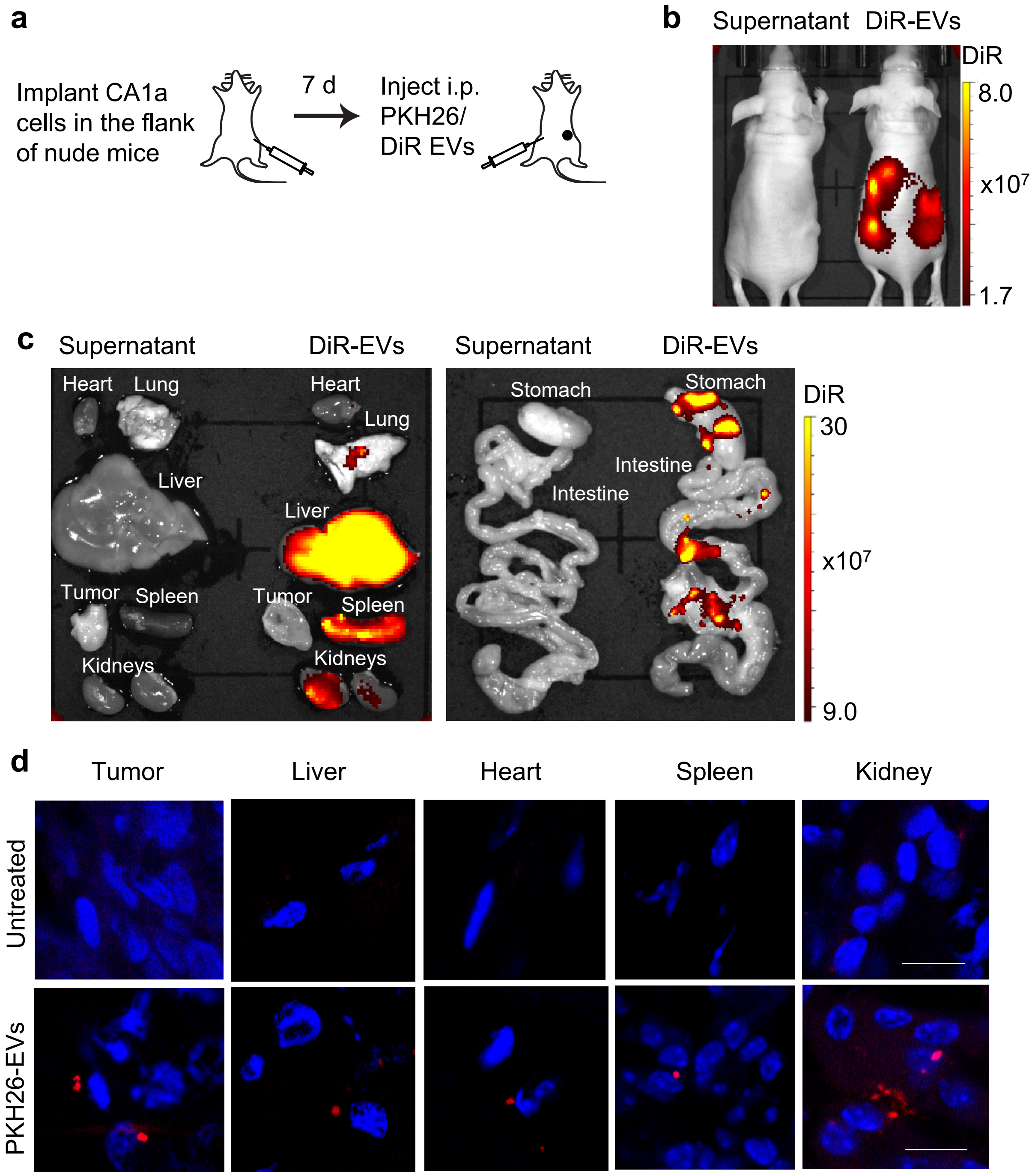

Supplementary Figure 12| Distribution of RBCEVs by systemic administration in nude mice. (a) Schematic presentation of the experiment: nude mice with small CA1a tumors (7 mm in diameter) were injected i.p. with 16.5 x 10^{11} PKH26-labeled or DiR-labeled RBCEVs. **(b)** Representative image of the live mice and **(c)** Representative ex vivo image of the organs from nude mice injected with DiR-labeled RBCEVs or the supernatant of the EV wash at 24 hours post-treatment. DiR fluorescence is presented as pseudocolored radiance (photon/s). **(d)** Cryosections of the organs with PKH26 fluorescence (red) and DAPI staining of the nuclei (blue) from nude mice injected with PKH26-labeled RBCEVs. Scale bar, 10 µm.

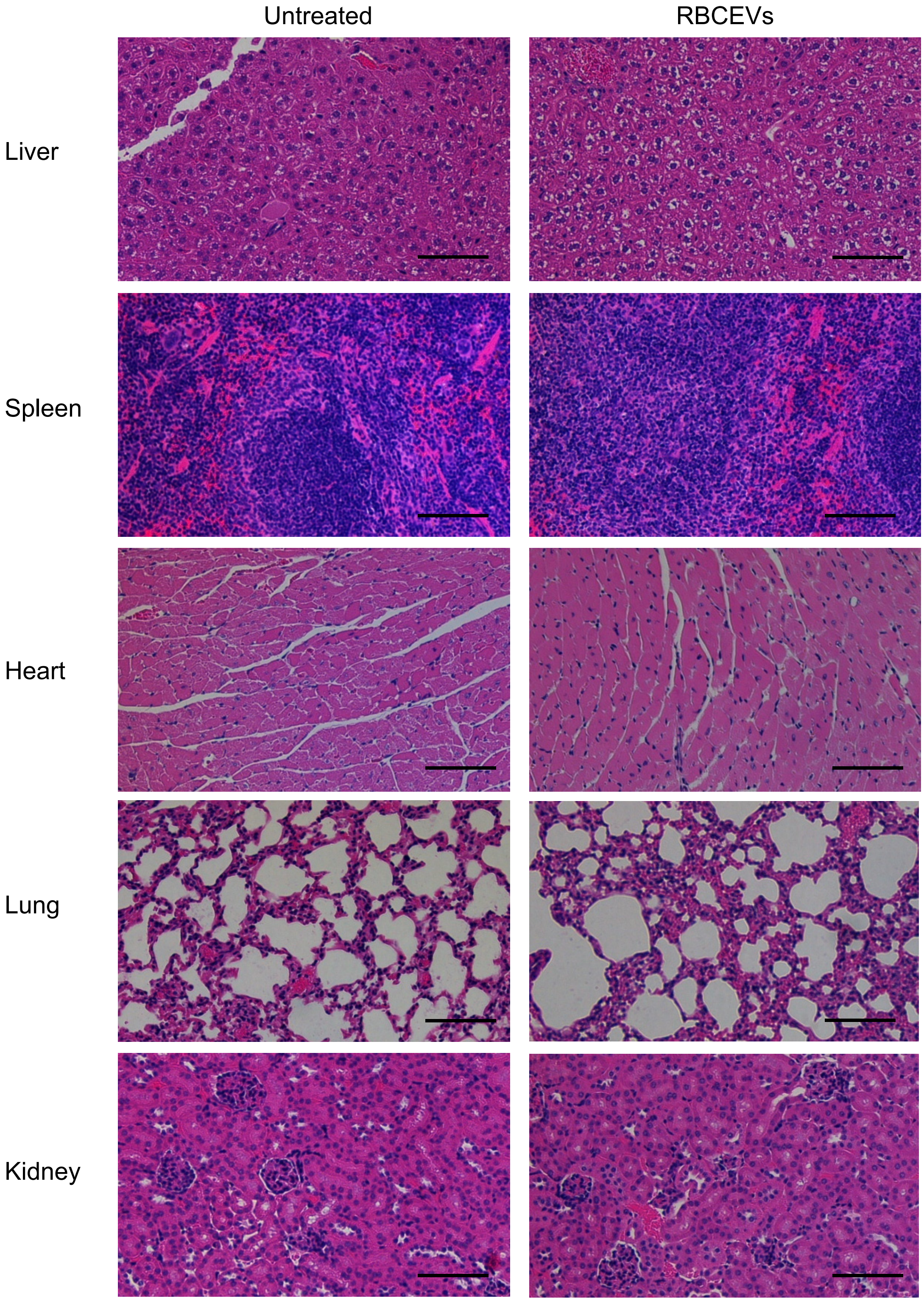

Supplementary Figure 13| RBCEV treatments do not affect the organs. Representative pictures of tissue sections stained with H & E from untreated and intraperitoneally PKH26-RBCEVs injected mice (as in Supplementary Fig. 12). Scale bar, 100 µm. The same morphology was observed in other samples (3 mice/group).

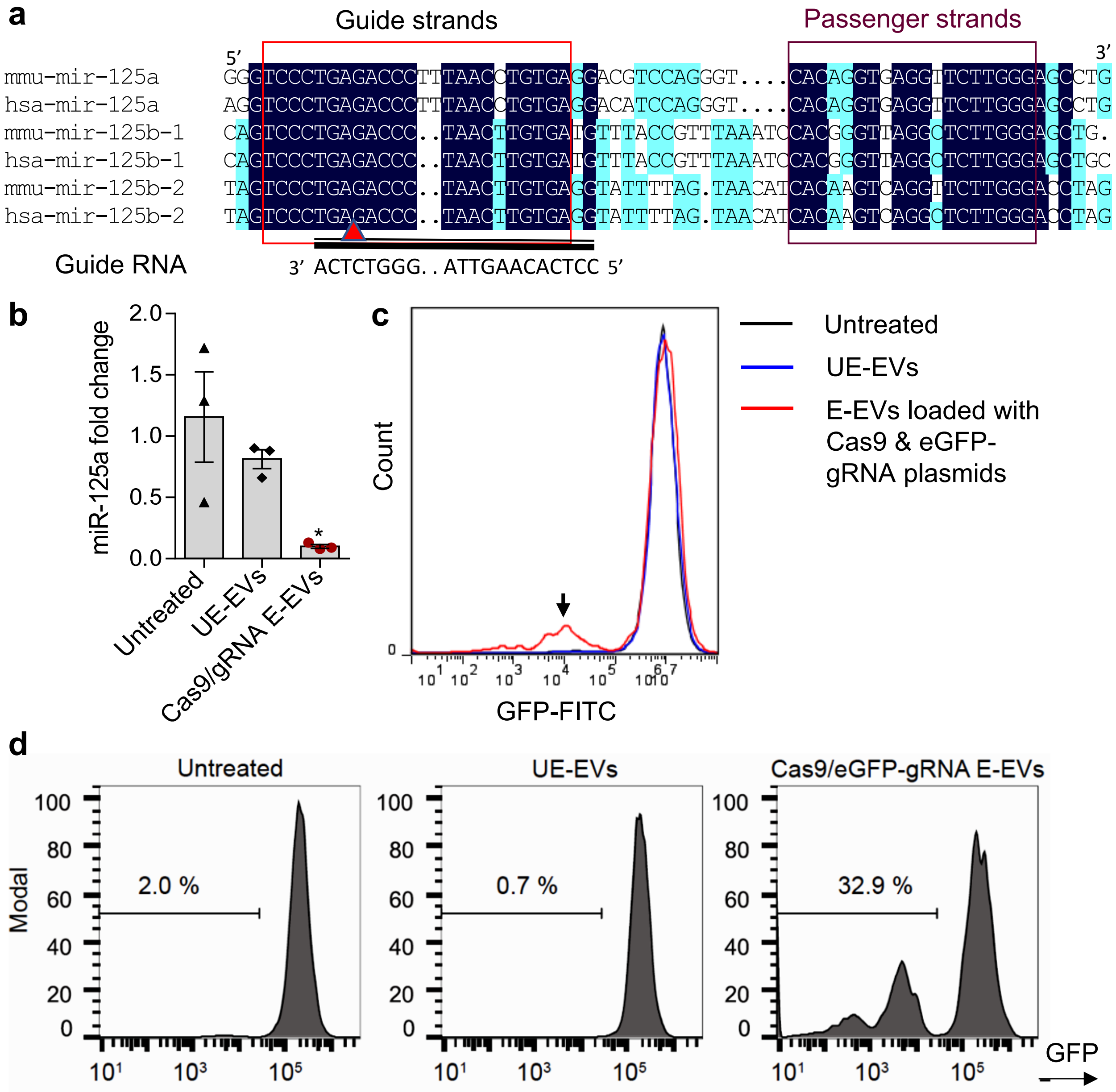

Supplementary Figure 14| RBCEVs deliver *Cas9* mRNA and gRNAs. (a) Sequences of *mir-125* loci in the human genome and the design of gRNA targeting these loci. Sequences were colored by their similarity (black: all identical; blue: half identical) using DNAMAN sequence analysis software. Guide strands are the major strands that are processed into the mature miR-125a or 125b of the miR-125 family. Guide RNA was designed such that mutations may occur in the seed sequence of mature miR-125s (arrow head). **(b)** Expression of miR-125a in MOLM13 cells treated with unelectroporated EVs (UE-EVs) or with EVs that were electroporated with *Cas9* mRNA and *mir-125b*-targeting gRNA for 48 hours (mean ± SEM; n = 3 cell passages). *P < 0.05, student's one-way t-test. **(c)** FACS analysis of GFP in 293T-eGFP cells incubated with UE-EVs or with EVs that were electroporated with *Cas9* plasmid and eGFP-targeting gRNA plasmid. GFP negative cells are indicated by the arrow. **(d)** FACS analysis of GFP expression in NOMO1-eGFP cells that were treated with UE-EVs or EVs loaded with *Cas9* mRNA and anti-eGFP gRNA for 7 days.

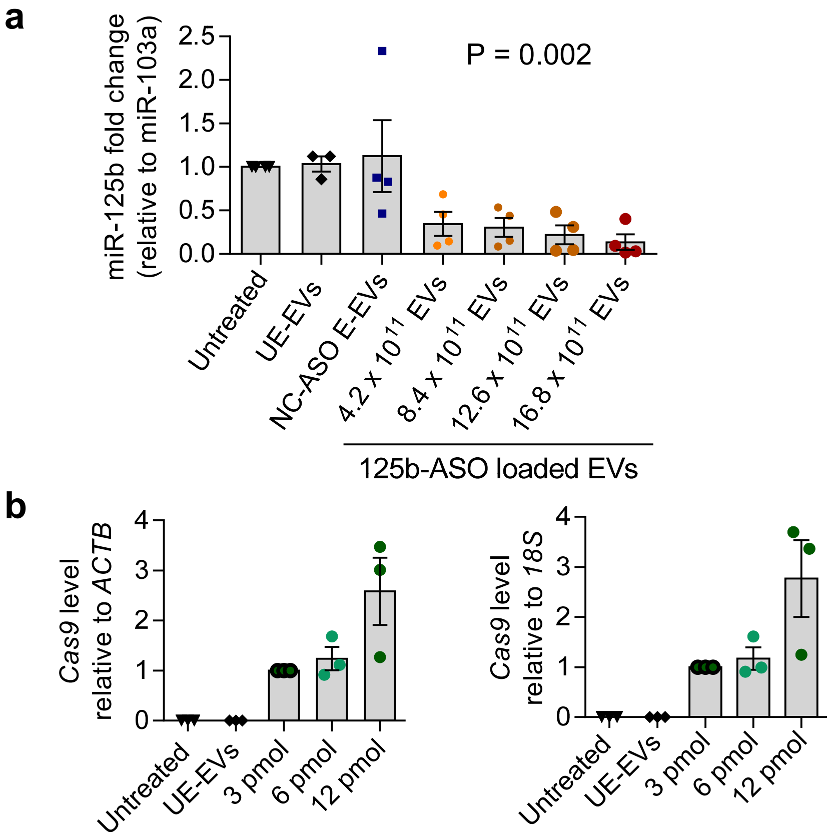

Supplementary Figure 15| Supplementary qPCR data with additional internal controls. (a) Expression fold change of miR-125b relative to miR-103a in MOLM13 cells that were untreated, incubated with 16.8 x 10^{11} unelectroporated RBC EVs (UE-EVs), with 16.8 x 10^{11} NC-ASO electroporated RBC EVs (E-EVs) or 4.2 – 16.8 x 10^{11} 125b-ASO-electroporated RBCEVs for 72 hours, determined using miRCURY-LNA qRT-PCR (mean ± SEM, n = 3 cell passages). One-way Anova test result is shown in the graph. **(b)** The level of *Cas9* mRNA relative to *ACTB* and *18S* RNA in MOLM13 cells that were incubated with 12.4 x 10^{11} unelectroporated RBCEVs (UE-EVs) or 12.4 x 10^{11} RBCEVs electroporated with 3, 6 or 12 pmol *Cas9* mRNA (E-EVs) after 24 hours of treatment, relative to the 3 pmol condition (mean ± SEM; n = 3 cell passages).

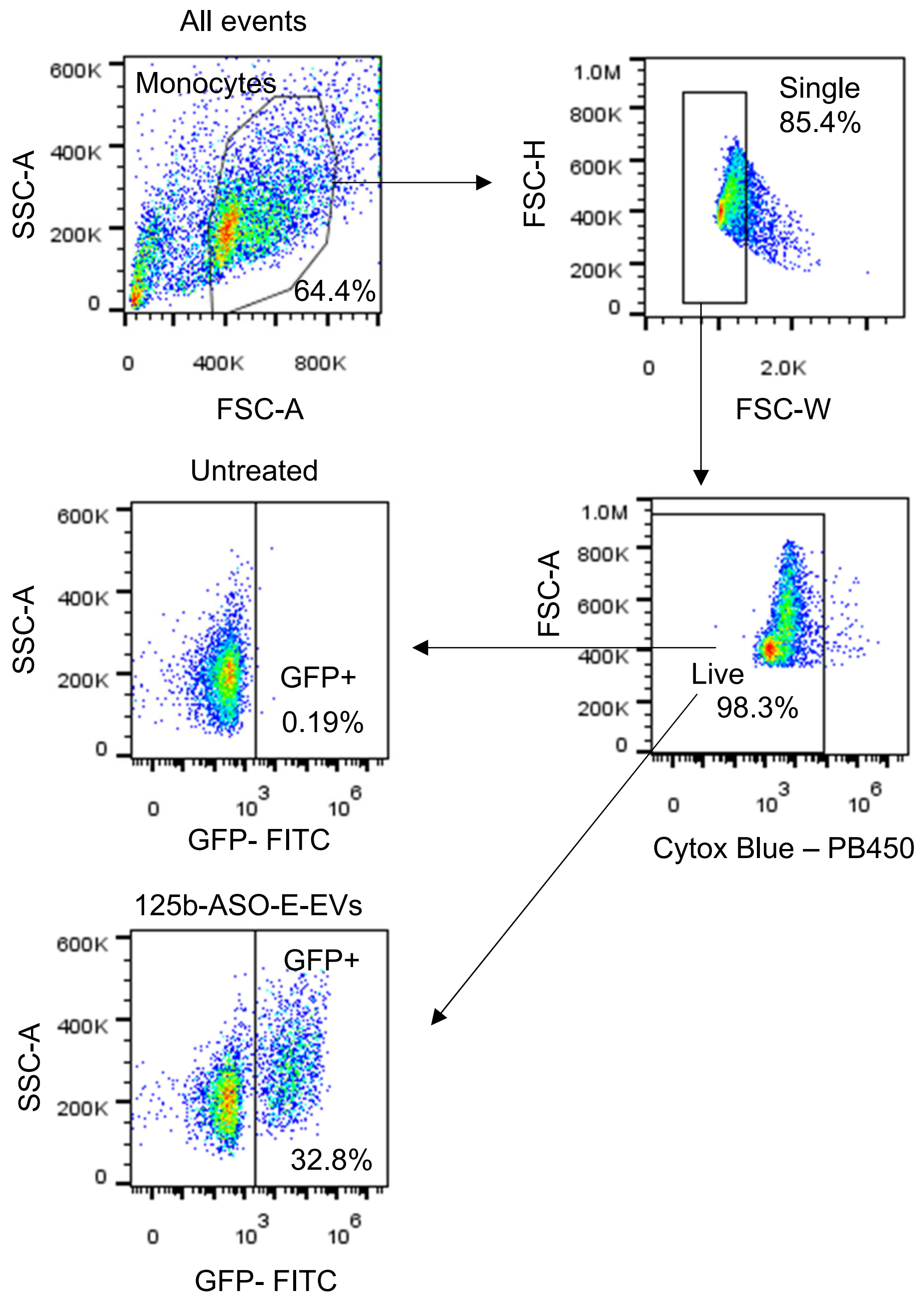

Supplementary Figure 16| Gating strategy for FACS analysis of bone marrow cells. FACS analysis of GFP cells in the bone marrow of NSG mice treated with 3.3 x 10^{12} RBCEVs containing 125b-ASO as in Fig. 7. Monocytes were gated based on FSC-A and SSC-A to exclude the debris, dead cells and RBCs (low FSC-A). The single cells were further gated from monocytes based on FSC-width vs. FSC-height, to exclude doublets and aggregates. The live cells were gated from the single cells population based on Cytox blue negative (PB450 channel). Subsequently, the GFP-positive cells were gated in FITC channel as the population that exhibit negligible GFP signals in the untreated negative control. The same gates were applied to all samples from the same batch.

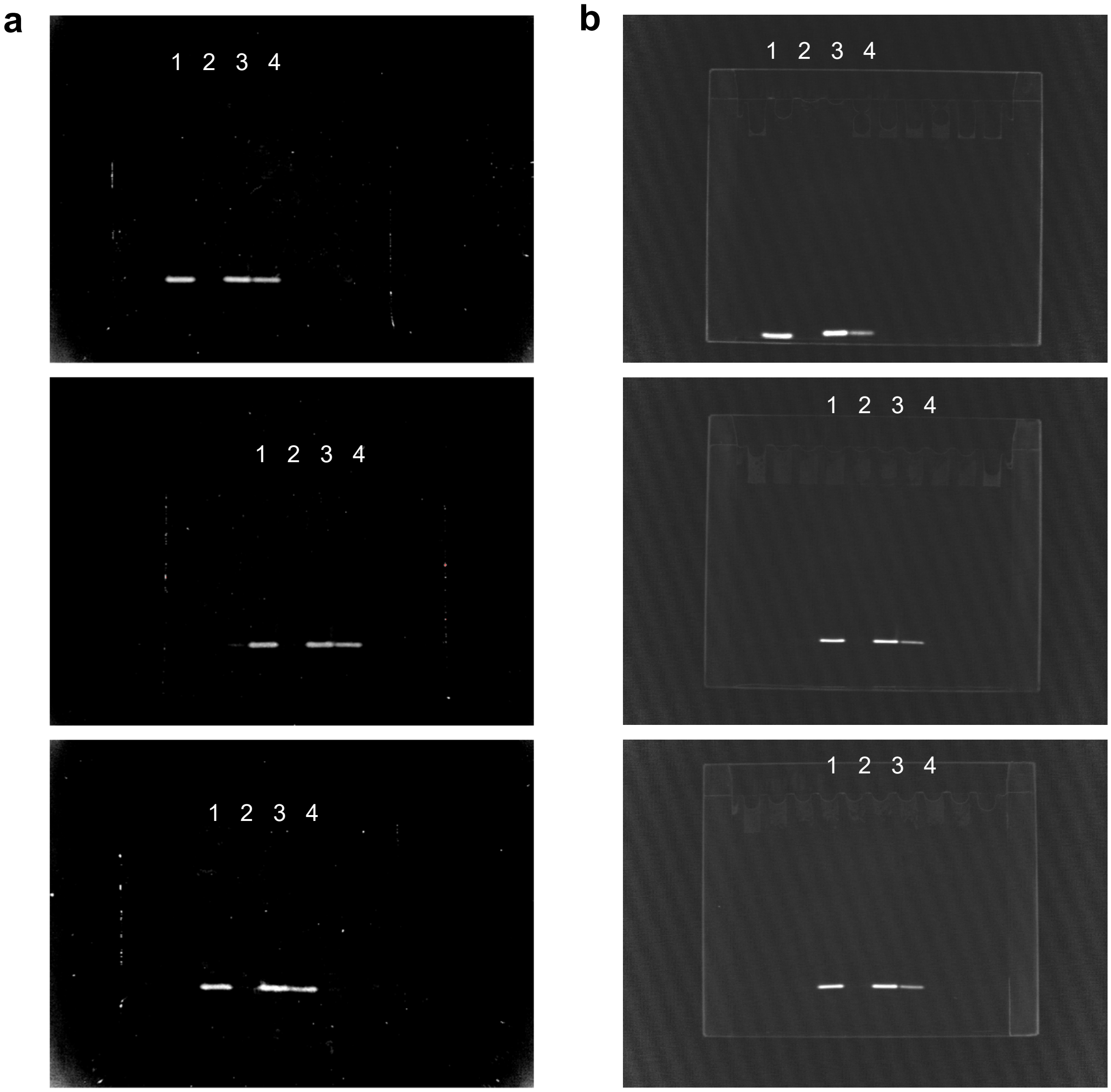

Supplementary Figure 17| Full images of the native gel electrophoresis.
(a) Separation of unlabeled NC-ASOs (22 bp): 200 pmol unlabeled NC-ASOs (lane 1), 8.25 x 10^{11} unelectroporated RBCEVs (lane 2), mixture of 200 pmol NC-ASOs and 8.25 x 10^{11} unelectroporated RBCEVs (lane 3), mixture of 200 pmol NC-ASOs and 8.25 x 10^{11} RBCEVs after electroporation (lane 4) loaded in 10% native gel and visualized using SYBR Gold staining in a Gel Doc^{TM} EZ Documentation system. **(b)** Separation of FAM-labeled NC-ASOs (22 bp): 200 pmol FAM NC-ASOs (lane 1), 8.25 x 10^{11} unelectroporated RBCEVs (lane 2), mixture of 200 pmol FAM NC-ASOs and 8.25 x 10^{11} unelectroporated RBCEVs (lane 3), mixture of 200 pmol FAM NC-ASOs and 8.25 x 10^{11} RBCEVs after electroporation (lane 4) loaded in 10% native gel and visualized by FAM fluorescence, using a Gel Doc^{TM} EZ Documentation system.

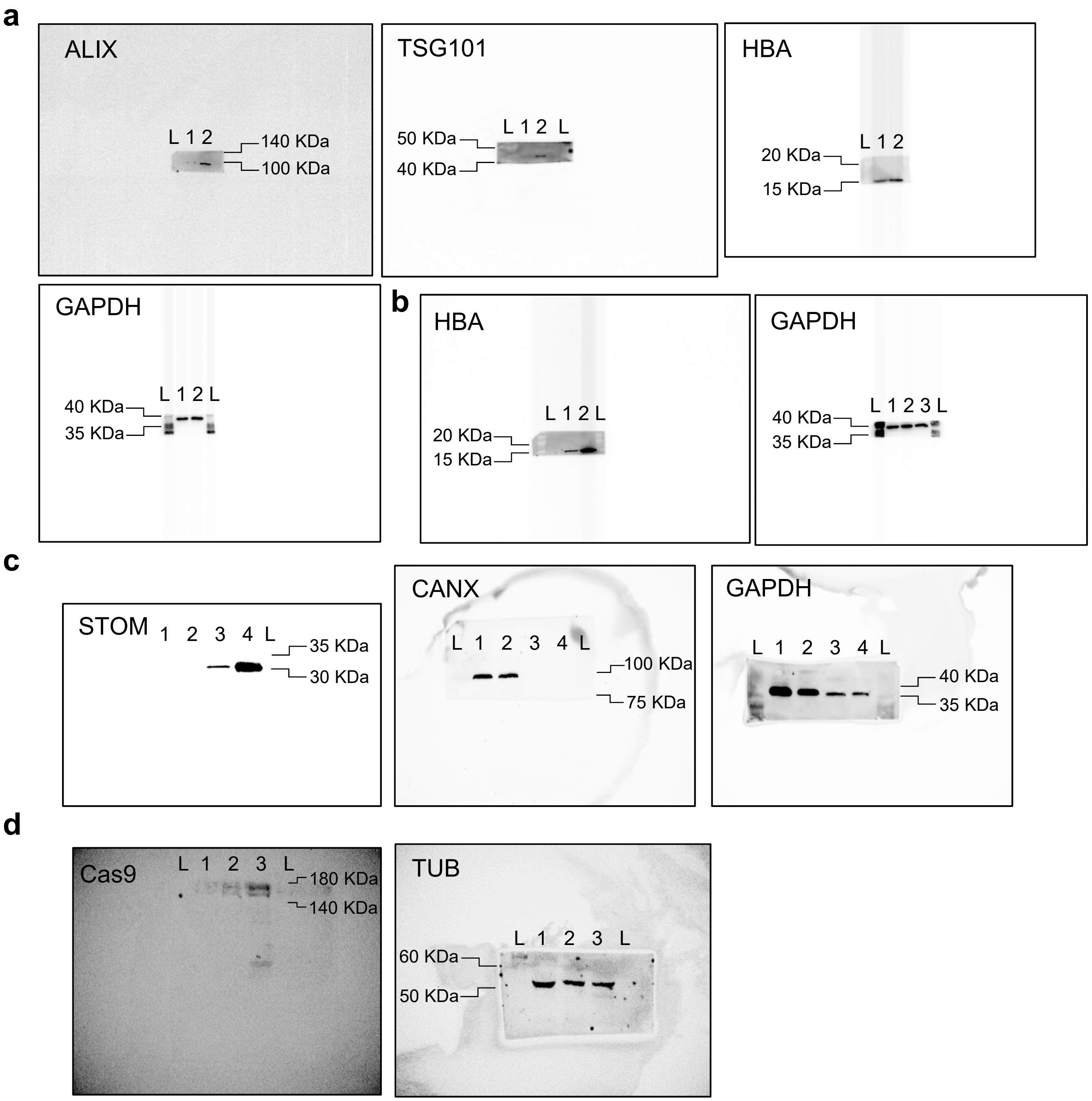

Supplementary Figure 18| Full images of the Western blots. (a) Western blot analysis of ALIX, TSG101 and HBA relative to GAPDH (loading control) in cell lysates (lane 1) and EVs (lane 2) from RBCs. **(b)** Western blot analysis of HBA and GAPDH in MOLM13 cells untreated (lane 1) or incubated with 8.25 x 10^{11} unelectroporated RBCEVs (lane 2) or with 8.25 x 10^{11} electroporated RBCEVs (lane 3) for 24 hours. **(c)** Western blot analysis of Stomatin (STOM), Calnexin (CANX), and GAPDH in leukemia MOLM13 cells (lane 1), NOMO1 cells (lane 2), RBCs (lane 3) and RBCEVs (lane 4). **(d)** Western blot analysis of Cas9 and TUB in MOLM13 cells untreated (lane 1), treated with unelectroporated RBCEVs (lane 2) or *Cas9* mRNA–loaded RBCEVs (lane 3). In all panels, the blots were cut horizontally and hybridized with multiple antibodies at the same time. Protein ladder (L) was loaded at 2 sides of the samples to determine the molecular weights.
